# HIV status, knowledge, attitudes and behaviour of persons with and without disability in South Africa: evidence from a national population-based survey

**DOI:** 10.11604/pamj.2019.33.302.17215

**Published:** 2019-08-19

**Authors:** Supa Pengpid, Karl Peltzer

**Affiliations:** 1 ASEAN Institute for Health Development, Mahidol University, Salaya, Thailand; 2 Department of Research Administration and Development, University of Limpopo, Polokwane, South Africa; 3 Department for Management of Science and Technology Development, Ton Duc Thang University, Ho Chi Minh City, Vietnam; 4 Faculty of Pharmacy, Ton Duc Thang University, Ho Chi Minh City, Vietnam

**Keywords:** Disability, HIV infection, HIV knowledge, population survey, South Africa

## Abstract

**Introduction:**

People with disabilities have been identified as a key risk population for HIV. The aim of this study was to investigate HIV status, knowledge, attitudes, behaviours and its correlates in persons with and without disabilities in South Africa.

**Methods:**

Cross-sectional data of 26404 participants 15 years and older from the "2012 South African national HIV prevalence, incidence and behaviour survey" were analysed.

**Results:**

1348(5.3%) had a disability. Persons with a disability were older (median age 52 years, IQR=24; versus 36 years, IQR=29), more often men, had a lower education and lower income and more likely living in a rural area than persons without disability. The prevalence of HIV infection was 16.7% in persons with disability, 23.0% in persons with visual/hearing or speech disability, 31.6% in persons with hearing disability and 16.2% in persons without disability. Antiretroviral (ARV) exposure in the HIV positive population was 41.3% among persons with disability and 30% in persons without disability. In multivariable logistic regression analysis, persons with disability had a lower odds to know an HIV testing site (Odds Ratio=OR: 0.46, Confidence Interval=CI: 0.22, 0.98) and a higher odds to have had two or more sexual partners in the past 12 months (OR 2.74, CI: 1.44, 5.21), had casual or transactional sex (OR: 6.25, CI: 2.57, 15.21) and psychological distress (OR: 2.10, CI: 1.50, 2.95) than persons without disability. In multivariable logistic regression analysis in both groups (with and without disability), psychological distress (OR: 2.90, CI: 1.53, 5.47, and OR: 1.90, CI: 1.20, 3.01, respectively) and high HIV stigma (OR: 0.31, CI: 0.25, 0.67, and OR: 0.57, CI: 0.34, 0.96, respectively) were associated with increased prevalence of HIV infection.

**Conclusion:**

The study found a high prevalence of HIV infection in persons with disabilities, in particular in those with hearing impairment. In some areas, persons with disability showed lower knowledge and higher risk behaviours than persons without disabilities. There is a need to strengthen HIV information and communication strategies geared towards targeting people with all types of disabilities.

## Introduction

"People with disabilities are at risk for exposure to HIV infection and are less likely to access prevention, testing and treatment" [1]. Several studies reported that persons with disabilities have lower levels of knowledge on HIV/AIDS [2-4], and engage more frequently in HIV sexual risk behaviour [3], such as more often involved transactional sex [5] and experienced increased risk of sexual violence [5]. Among people with disabilities in sub-Saharan Africa, the HIV prevalence varied across 13 studies from 1.1% to 29%, and the pooled rate of HIV infection in people with disabilities compared to the general population was "1.31 (1.02-1.69) overall; 1.16 (0.71-1.87) among people with mental illness or intellectual disabilities and 1.07 (0.58-1.95) among people with hearing disabilities" [6]. In a more recent study in Cameroon, De Beaudrap *et al*. [5] found that 3.9% in the control population had a positive HIV test result compared with 6.8% in people with disabilities. In Uganda, persons with disabilities also reported having a sexually transmitted disease (STD) within the last 12 months at significantly higher rates than peers without disabilities [2]. Among persons with disability, sexual violence and sex work were strongly associated with increased risk of HIV infection in Cameroon [5]. There is limited information on HIV status, knowledge, attitudes and behaviours and its correlates in Africa, including South Africa [1]. Therefore, the aim of this study was to investigate HIV status, knowledge, attitudes, behaviours and its correlates in persons with and without disabilities in South Africa.

## Methods

**Data and sampling:** cross-sectional data from the "2012 South African national HIV prevalence, incidence, and behaviour survey" were analysed [7]. The sampling strategy was stratified by province, type of geolocality and predominant population or racial groups. Using multistage sampling, a random sample of "enumeration areas" (EAs) was selected and within EAs households were randomly selected. All individuals within a household were eligible to participate. Trained and supervised field workers interview-administered a questionnaire and nurses collected dried blood spots (DBS) specimens. Informed consent was attained prior to the conduct of the interview and DBS collection. The detailed survey methods are described elsewhere [7]. This analysis is based on data on individuals aged 15 years and older who participated in the survey. The study survey proposal was approved by the "HSRC Research Ethics Committee (REC: 5/17/11/10)" and by the "Centers for Disease Control and Prevention" (CDC). The response rate for participating in the survey at the household level was 84.7% and for HIV testing 67.5%.

**Measures:** disability was assessed with the question, "Do you have any disability" and the type of disability: "physical (spinal injury, loss of limb, etc.); sight; hearing; communication or speech, and mental or psychiatric illness)" [7]. Socio-economic measures included age, educational level, gross monthly income, and area of residence [7]. HIV and antiretroviral (ARV) status. Dried blood spots (DBS) specimens were tested anonymously for HIV antibodies. Samples that tested positive for HIV-1 antibodies were tested for the presence of ARVs; more details [8]. HIV/AIDS knowledge was assessed with eight items, e.g., "Can AIDS be cured" Response options were "yes", "no" or "unsure" [7]. Only correct responses were scored with one and incorrect or unsure responses with zero, to arrive at a summary score; scores 6 or more were classified as high and scores 0-5 as low. Cronbach alpha of this scale was 0.69 in this sample. In addition, one item assessed knowledge of a place nearby where one get an HIV test.

HIV/AIDS stigma was measured with seven items, e.g., "If you knew that a shopkeeper or food seller had HIV, would you buy food from them" [7] Response options were "yes", "no" or "unsure." Only stigmatizing, including unsure, responses were scored with one and non-stigmatizing with zero, to arrive at a summary score; scores 3 or more were classified as high and scores 0-2 as low. Cronbach alpha of this scale was 0.58 in this sample. Sexual risk behaviour questions included, 1) "Have you had sex during the past 12 months" 2) "Overall, how many sexual partners did you have during the past 12 months" (Coded two or more=1 and 0 to 1 sexual partner=0). 3) "In the past six months, how many sex partners have you had whose HIV status you did not know at the time that you had sex" (Coded 1 or more=1 and none=0). 4) "Most recent person with whom you had sex in the past 12 months" (Responses ranged from 1=husband or wife to 4=casual partner and 5=someone whom you paid for sex) (Coded 1 or 4=1 and others=0). 5) "How often do you use a condom with this particular partner" (Inconsistent condom use was coded every time=0 and almost every time or sometimes=1) 6) "Did you use a condom last sex" (Yes, No) 7) "The last time you had sex, did you drink alcohol before sex" (Yes, No) Men were also asked, "Have you been circumcised" (Yes, No) [7].

Intimate partner violence. Participants who conceded that they were in an intimate relationship responded to five intimate partner violence questions, e.g. "In the past 12 months, a partner has hit me (with a fist or slap or something else that could hurt me)." "In the past 12 months, a partner has forced me to have sex against my wishes by using violence or threatening violence." (Response options were "yes" and "no") [7,9]. A positive response to any of the five questions was classified as intimate partner violence, and a positive response to the second question as sexual violence victimization. Psychological distress was measured with the 10-item "Kessler Psychological Distress Scale (K-10)", e.g., "In the past 30 days, how often did you feel so restless that you could not sit still" Response options ranged from 1= "none of the time" to 5= "all the time." These scores were added-up, with higher total scores indicating higher psychological distress [10]. A cut-off of 20 scores and more for detecting depression and anxiety disorders was used [10]. Cronbach alpha for the K-10 in this sample was 0.90. Hazardous or harmful alcohol use was assessed with the 10-item "Alcohol Disorder Identification Test (AUDIT)," e.g., "How often did you have a drink containing alcohol in the past 12 months" [11]. Response options range from 0 to 4, with a summed total range from 0 to 40 scores; a score of 8 or more indicated hazardous or harmful alcohol use [11]. Cronbach alpha for the AUDIT in this sample was 0.84.

**Data analysis:** descriptive statistics were used to summarize the prevalence of study variables. Pearson Chi-square was used to test for differences by disability status. Multivariable logistic regression was used to estimate the impact of disability status on HIV/AIDS knowledge, HIV/AIDS stigma, sexual risk behaviours and psychological distress. In addition, multivariable logistic regression was utilized to estimate associations with HIV positive status for persons with disability and persons without disability separately. All variables that were statistically significant at the P <0.05 levels in bivariate analyses were included in the multivariable model. In the paper, weighted percentages are presented. The "svy" command was utilized to take into account the multi-stage cluster design of the survey. All statistical analyses were performed utilizing Stata software version 12 (Stata Corp., College Station, TX, USA).

## Results

**Sample characteristics:** from the total sample of 26404 participants 15 years and older, 1348 (5.3%) had a disability. Persons with a disability were older (median age 52 years, IQR=24; versus 36 years, IQR=29), more often men, had a lower education and lower income and more likely living in a rural area than persons without disability (Table 1).

**Table 1:** sample characteristics by disability status

Variable	Persons with disability	Persons without disability	P-value
**All Age in years**	1348 (5.3)	25056 (94.7)	<0.001
15-29	195 (17.8)	9840 (41.7)	
30-44	273 (29.0)	6420 (30.9)	
45 or more	880 (53.0)	8796 (27.4)	
**Sex**			<0.001
Female	701 (42.4)	14267 (52.4)	
Male	647 (57.6)	10789 (47.6)	
**Education**			<0.001
Grade 0-7	382 (37.9)	3856 (17.5)	
Grade 8-11	393 (39.7)	8963 (42.8)	
Grade 12 or more	229 (22.4)	8592 (39.7)	
**Income in Rand**			<0.001
0-1199	364 (40.1)	4223 (36.7)	
1200-2999	472 (46.4)	4302 (29.1)	
3000 or more	126 (13.5)	4913 (34.3)	
**Residence**			0.004
Urban formal	739 (43.6)	14851 (52.6)	
Urban informal	153 (8.3)	2522 (8.2)	
Rural formal	352 (43.3)	5227 (33.7)	
Rural informal	104 (4.8)	2455 (5.5)	

**HIV status and risk variables by disability status:** the prevalence of HIV infection was 16.7% in persons with disability, 23.0% in persons with visual/hearing or speech disability, 31.6% in persons with hearing disability and 16.2% in persons without disability. ART exposure in the HIV positive population was 41.3% among persons with disability and 30% in persons without disability. Compared to persons without disability, persons with disability had significantly lower HIV/AIDS knowledge, less likely knew an HIV testing site and less likely having not used a condom at last sex. Persons with disability had significantly higher HIV/AIDS stigma attitudes, had two or more sexual partners in the past 12 months, had casual or transactional sex, and psychological distress than persons without disability (Table 2). In multivariable logistic regression analysis, persons with disability had a lower odds to know an HIV testing site (Odds Ratio=OR: 0.46, Confidence Interval=CI: 0.22, 0.98) and a higher odds to have had two or more sexual partners in the past 12 months (OR 2.74, CI: 1.44, 5.21), had casual or transactional sex (OR: 6.25, CI: 2.57, 15.21), and psychological distress (OR: 2.10, CI: 1.50, 2.95) than persons without disability (Table 3).

**Table 2:** HIV status and risk variables by disability status in 15 years and older persons

Variable	Persons with disability	Persons without disability	P-value	Physical disability	Visual/hearing/speech disability	Mental disability
	N=1348	N=25056		N=627	N=422	N=175
	N (%)	N (%)		N (%)	N (%)	N (%)
HIV positive	134 (16.7)	2439 (16.2)	0.832	66 (18.0)	43 (23.0)	19 (9.8)
ARV positive	58 (41.3)	744 (30.0)	0.092	31 (38.8)	19 (45.1)	5 (13.1)
HIV/AIDS knowledge (high)	442 (33.8)	10465 (41.6)	0.008	235 (38.3)	117 (29.5)	45 (29.2)
HIV/AIDS stigma (high)	546 (38.8)	7411 (25.9)	<0.001	233 (32.8)	184 (38.4)	89 (51.2)
Know HIV testing site	1127 (84.3)	22611 (93.2)	<0.001	539 (83.2)	343 (84.4)	130 (76.0)
Two or more sexual partners in the past 12 months	49 (19.0)	1424 (12.5)	0.031	30 (18.2)	10 (18.7)	3 (12.6)
Last sexual partner (casual or transactional)	17 (6.5)	381 (2.4)	0.019	13 (8.0)	3 (6.9)	1 (1.4)
No condom last sex	41 (14.3)	1328 (23.6)	0.016	17 (15.2)	10 (7.5)	8 (20.7)
Inconsistent condom use	417 (71.4)	11258 (73.0)	0.672	226 (80.1)	112 (58.8)	30 (61.9)
Alcohol use at last sex	54 (8.5)	916 (7.4)	0.597	32 (9.1)	10 (7.7)	5 (6.1)
Sex with unknown HIV status	102 (31.6)	2701 (28.8)	0.536	48 (32.8)	29 (23.6)	10 (41.4)
Male circumcision	241 (43.4)	4227 (47.2)	0.379	107 (40.1)	78 (50.3)	33 (25.7)
Intimate partner violence victimization	36 (7.0)	867 (7.2)	0.918	15 (7.4)	7 (5.0)	10 (11.4)
Sexual violence victimization	7 (1.1)	164 (1.5)	0.658	3 (1.4)	2 (1.0)	2 (1.0)
Psychological distress	490 (39.1)	5021 (23.7)	<0.001	229 (39.2)	148 (40.4)	81 (44.4)
Hazardous or harmful alcohol use	138 (12.4)	2403 (11.0)	0.474	69 (10.7)	37 (15.3)	15 (12.4)

**Table 3:** multivariable analysis: HIV knowledge, attitudes and behaviour

Outcome variable with disability as main explanatory variable	AOR (95% CI)^1^	P-value
HIV/AIDS knowledge (high)	0.98 (0.79, 1.38)	0.923
HIV/AIDS stigma (high)	1.20 (0.85, 1.70)	0.292
Know HIV testing site	0.46 (0.22, 0.98)	0.043
Two or more sexual partners in the past 12 months	2.74 (1.44, 5.21)	0.002
Last sexual partner (casual or transactional)	6.25 (2.57, 15.21)	<0.001
No condom use at last sex	0.56 (0.26, 1.24)	0.153
Psychological distress	2.10 (1.50, 2.95)	<0.001

AOR=Adjusted odds ratio; ^1^Adjusted for age, sex, education, income and residence type

**Associations with HIV status by disability status:** in multivariable logistic regression analysis in both groups (with and without disability), psychological distress (OR: 2.90, CI: 1.53, 5.47, and OR: 1.90, CI: 1.20, 3.01, respectively) and high HIV stigma (OR: 0.31, CI: 0.25, 0.67, and OR: 0.57, CI: 0.34, 0.96, respectively) were associated with increased prevalence of HIV infection. Middle age (OR: 3.11, CI: 2.08, 4.67), being female (OR: 0.63, CI: 0.42, 0.96), and having used a condom at last sex (OR: 2.60, CI: 1.66, 4.77) were associated with higher HIV prevalence in the controls (those without disability) but not in participants with disability (Table 4).

**Table 4:** associations with HIV positive status by disability status

Variable	Persons with disability		Person without disability	
	UOR (95% CI)	AOR (95% CI)	UOR (95% CI)	AOR (95% CI)
**Age in years**				
15-29	1 (Reference)	---	1 (Reference)	1 (Reference)
30-44	2.31 (0.95, 5.64)		2.86 (2.42, 3.38)***	3.11 (2.08, 4.67)***
45 or more	0.55 (0.23, 1.31)		0.79 (0.67, 0.94)**	1.75 (0.89, 3,44)
**Sex**				
Female	1 (Reference)	---	1 (Reference)	1 (Reference)
Male	1.02 (0.54, 1.93)		0.63 (0.54, 0.74)***	0.63 (0.42, 0.96)*
**Education**				
Grade 0-7	1 (Reference)		1 (Reference)	1 (Reference)
Grade 8-11	1.48 (0.67, 3.24)	---	0.90 (0.75, 1.09)	1.06 (0.61, 1.85)
Grade 12 or more	0.59 (0.24, 1.48)		0.62 (0.50, 0.77)***	0.62 (0.35, 1.10)
**Income in Rand**				
0-1199	1 (Reference)	---	1 (Reference)	1 (Reference)
1200-2999	1.53 (0.68, 3.46)		0.93 (0.75, 1.14)	1.18 (0.84, 1.95)
3000 or more	1.86 (0.65, 5.32)		0.45 (0.34, 0.59)***	0.65 (0.41, 1.03)
Intimate partner violence victimization	0.48 (0.06, 3.54)	---	1.57 (1.15, 2.15)**	1.28 (0.71, 2.30)
Hazardous or harmful alcohol use	1.05 (0.34, 3.21)	---	0.84 (0.66, 1.08)	---
Psychological distress (20 or more)	2.80 (1.52, 5.14)***	2.90 (1.53, 5.47)***	1.79 (1.52, 2.11)***	1.90 (1.20, 3.01)**
**HIV/AIDS knowledge**		---		
High	1 (Reference)		1 (Reference)	1 (Reference)
Low	1.27 (0.65, 2.47)		0.85 (0.72, 0.99)*	0.91 (0.61, 1.36)
**HIV/AIDS stigma**				
High	1 (Reference)		1 (Reference)	1 (Reference)
Low	0.43 (0.21, 0.86)*	0.31 (0.15, 0.67)**	0.73 (0.61, 0.87)***	0.57 (0.34, 0.96)*
Two or more sexual partners in the past 12 months	1.37 (0.48, 3.92)	---	0.95 (0.70, 1.29)	---
Condom use at last sex	1.49 (0.41, 5.42)		1.64 (1,20-2.09)***	2.60 (1.66, 4.07)***

UOR=Unadjusted odds ratio; AOR=Adjusted odds ration;

*** P<0.001;

** P<0.01;

* P<0.05

## Discussion

The study aimed at investigating HIV status, knowledge, attitudes and behaviours and its correlates in persons with and without disabilities in South Africa. The study found a high prevalence of HIV infection in persons with disabilities (16.7%), in particular in those with hearing impairment (31.6%). In the review of HIV prevalence among people with disabilities in sub-Saharan Africa, people with disabilities and in particular hearing disabilities were not found to have a lower risk for HIV infection than people without disabilities [6] and in a study in Cameroon at higher risk [5]. In the case of hearing disabilities, the review [6] notes that they have more often-inaccurate HIV knowledge. This study confirmed that persons with hearing impairment had together with persons with communication and speech impairment (21.6% and 5.9% high HIV/AIDS knowledge, respectively) had the poorest HIV/AIDS knowledge among the different types of disabilities. Consistent with a previous study [5], this study shows that people with disabilities have additional disadvantages such as lower education and lower income compared to persons with disabilities, which makes them even more vulnerable to HIV risk. Consistent with previous studies [2-4], this study found in bivariate analysis that persons with disabilities had lower levels of knowledge on HIV/AIDS than persons without disabilities. Also, in agreement with several studies [3,5], this study found that persons with disabilities engaged more often in HIV sexual risk behaviours, such as having multiple sexual partners and casual or transactional sex, than their counterparts without disabilities. The finding that individuals with a disability experienced more psychological distress than in persons without disability may refer to the additional mental distress people with disability suffer from due to their impairments. This study found that in both groups (with and without disability), psychological distress and high HIV stigma were associated with increased prevalence of HIV infection. This finding emphasises the need for mental health and stigma prevention interventions. While sexual violence was associated with increased risk of HIV infection in Cameroon [5], this study could not find such an association. These differences may be attributed to different ways of assessing sexual violence.

**Study strength and limitations:** the study included a very large community sample of persons with and without disabilities with assessments of HIV status, ARV exposure and HIV/AIDS knowledge, attitudes and behabiour. Disability was only assessed with one item, which has its limitations. Future studies may want to include a more comprehensive assessment, such as using the World Health Organization Disability Assessment Schedule 2.0 (WHODAS 2.0). Due to the cross-sectional nature of the study no causative conclusions can be drawn.

## Conclusion

The study found a high prevalence of HIV infection in persons with disabilities, in particular in those with hearing impairment. In some areas, persons with disability showed lower knowledge and higher risk behaviours than persons without disabilities. There is a need to strengthen HIV information and communication strategies geared towards targeting people with all types of disabilities.

### What is known about this topic

- People with disabilities are at risk for exposure to HIV infection and are less likely to access prevention, testing and treatment;
- In persons with disabilities the HIV prevalence varied across 13 studies in sub-Saharan Africa from 1.1% to 29%.

### What this study adds

- The current (2012) study showed an HIV prevalence of 16.7% in persons with disability in South Africa;
- Persons with disability had lower odds to know an HIV testing site and a higher odds to have had two or more sexual partners in the past 12 months, had casual or transactional sex and psychological distress than persons without disability;
- In both groups (with and without disability), psychological distress and high HIV stigma were associated with increased prevalence of HIV infection

## Competing interests

The authors declare no competing interest.
